# Comparative Analysis of Extracellular Vesicle Isolation From Equine Serum and Plasma Using Two Isolation Methods With Structural and Proteomic Validation

**DOI:** 10.1096/fj.202504053R

**Published:** 2026-01-18

**Authors:** Dominika Milczek‐Haduch, Magdalena Żmigrodzka, Paula Kiełbik, Bianka Świderska, Jacek Olędzki, Olga Witkowska‐Piłaszewicz

**Affiliations:** ^1^ Department of Large Animals Diseases and Clinic, Institute of Veterinary Medicine Warsaw University of Life Sciences Warsaw Poland; ^2^ Department of Morphological Sciences, Institute of Veterinary Medicine Warsaw University of Life Sciences Warsaw Poland; ^3^ Department of Pathology and Veterinary Diagnostic, Institute of Veterinary Medicine Warsaw University of Life Sciences Warsaw Poland; ^4^ Mass Spectrometry Laboratory Institute of Biochemistry and Biophysics, Polish Academy of Sciences Warsaw Poland

**Keywords:** exosomes, extracellular vesicles, horses, nanoparticles, plasma, serum

## Abstract

Extracellular vesicles (EVs) are promising biomarkers and mediators of intercellular communication, but their isolation from equine biofluids remains challenging. This study compared two isolation workflows—size‐exclusion chromatography (SEC) and differential ultracentrifugation followed by SEC (UC + SEC)—to evaluate their efficiency, reproducibility, and the proteomic composition of EVs derived from equine serum and plasma. Blood from six healthy horses was processed to obtain platelet‐free plasma and serum. EVs were isolated using SEC or UC + SEC and characterized by transmission electron microscopy, nanoparticle tracking analysis, and mass spectrometry‐based proteomics with functional enrichment analysis. SEC provided higher reproducibility, greater protein yield, and a simpler workflow than UC + SEC. Serum combined with SEC yielded the most consistent proteomic profiles, with strong detection of typical EV markers and the largest overlap among replicates. Vesicles displayed the expected morphology and size distribution, with a predominant population between 100 and 200 nm. Plasma‐derived EVs were enriched in proteins related to translation, chaperone activity, and proteasome function, while serum‐derived EVs contained proteins involved in immune processes, cytoskeletal organization, adhesion, and hemostasis. Both the isolation method and biological matrix significantly influenced EV yield and proteome composition. SEC applied to serum provided a reproducible and high‐quality EV preparation suitable for biomarker discovery. As this was a methodological comparison in healthy animals, diagnostic test performance metrics (sensitivity, specificity, PPV/NPV) were outside the scope of the study; our goal was to quantify upstream analytical determinants (matrix and isolation workflow) that influence reproducibility and discovery‐phase proteomic readouts.

## Graphical abstract

Schematic workflow of plasma‐ and serum‐derived extracellular vesicle (EV) analysis in horses, including sample preparation, EV isolation using size‐exclusion chromatography (SEC) and ultracentrifugation (UC), vesicle characterization by nanoparticle tracking analysis (NTA) and transmission electron microscopy (TEM), and comparative proteomic profiling revealing distinct functional enrichments in plasma‐ and serum‐derived EVs.

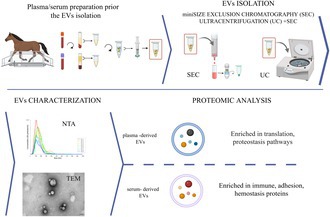

**Abbreviations**
ACD: acid–citrate–dextrose
AGC: automatic gain control
BP: biological process
CC: cellular component
DDA: data‐dependent acquisition
dPBS: Dulbecco's phosphate‐buffered saline
EDTA: ethylenediaminetetraacetic acid
EV: extracellular vesicle
EV markers: tetraspanins CD9, CD63, CD81, CD82, TSPAN7, TSPAN9, TSG101, PDCD6IP
FA: formic acid
FDR: false discovery rate
GO: gene ontology
HDL: high‐density lipoprotein
ISEV: international society for extracellular vesicles
KEGG: Kyoto Encyclopedia of Genes and Genomes
LC–MS/MS: liquid chromatography–tandem mass spectrometry
LDL: low‐density lipoprotein
MF: molecular function
MMTS: methyl methanethiosulfonate
MS: mass spectrometry
MS/MS: tandem mass spectrometry
NTA: nanoparticle tracking analysis
OA: osteoarthritis
PBS: phosphate‐buffered saline
PFP: platelet‐free plasma
PSM: peptide‐spectrum match
QC: quality control
RT: room temperature
SEC: size‐exclusion chromatography
sEV: small extracellular vesicle
SP3: single‐pot, solid‐phase‐enhanced sample preparation
TEM: transmission electron microscopy
UC: ultracentrifugation
UC + SEC: differential ultracentrifugation followed by size‐exclusion chromatography
WP: WikiPathways

## 1 Background

Extracellular vesicles (EVs) are nano‐sized particles released by cells into the extracellular space. They are increasingly recognized as key mediators of intercellular communication and as promising biomarkers across human and veterinary medicine. Among available biofluids, blood is a particularly rich and accessible source of EVs with substantial diagnostic and prognostic potential. Blood‐derived EVs are a key substrate for translational biomarker development, as they allow minimally invasive, repeatable sampling and reflect systemic physiological and pathophysiological states. Blood EV profiles have emerged as a central substrate for the discovery of minimally invasive biomarkers with serum/plasma EV cargo increasingly evaluated across disease areas, including oncology and neurodegeneration [1, 2, 3]. However, in horses specifically, robust evidence on optimal methods for obtaining and processing blood for EV analysis remains sparse and inconsistent, limiting reproducibility and clinical translation. Optimizing protocols for EV isolation from equine blood is particularly important due to the limited data available in veterinary medicine [4].

EV measurements are highly sensitive to preanalytical and methodological variables—including blood collection technique, hemolysis, the choice of serum versus plasma, anticoagulant type, freeze–thaw cycles, storage conditions, and the isolation method—each of which can shift EV yield, composition, and downstream readouts [5]. While such issues are increasingly addressed in human EV research, their magnitude and practical solutions are underexplored in veterinary applications; equine data, in particular, are limited and difficult to compare across studies [6, 7]. Although the International Society for Extracellular Vesicles (ISEV) has proposed minimal criteria spanning biochemical, biophysical, and functional characteristics to improve study reproducibility, the lack of broadly applicable markers across species and the absence of standardized isolation protocols continue to challenge equine EV research [5, 6, 7]. Importantly, these reporting and characterization principles are equally relevant to biomarker studies, because pre‐analytical variables and co‐isolated components can materially reshape EV profiles and thereby affect discovery‐stage reproducibility and downstream clinical translation.

Even the basic choice of starting material is unsettled. Serum is easy to obtain and widely used, but coagulation can generate platelet‐derived EVs that confound proteomic profiles. Plasma—collected with anticoagulants such as ethylenediaminetetraacetic acid (EDTA), sodium citrate, or acid–citrate–dextrose (ACD)—avoids coagulation‐induced artifacts, yet its preparation is more technically demanding and anticoagulants may interact with EVs, reducing detectability and skewing particle counts and cargo measurements [8]. Moreover, the extent to which coagulation and blood processing steps shape EV composition in horses—and how these factors intersect with the chosen isolation strategy—remains unclear [9].

Regarding isolation, size‐exclusion chromatography (SEC) and ultracentrifugation (UC), often used alone or in combination, are among the most widely adopted approaches [10]. Importantly, equine blood exhibits species‐specific features—including an HDL‐dominant lipoprotein profile and distinct platelet/hemostatic biology—that can materially affect EV isolation trade‐offs, necessitating species‐specific methodological benchmarking rather than direct extrapolation from human workflows [11, 12]. SEC efficiently separates vesicles from protein contaminants and soluble molecules, preserving vesicle morphology and functionality; it is gentle, cost‐effective, and scalable, but typically yields fewer particles and often requires post‐isolation concentration [13]. UC can provide larger EV quantities and remains accessible due to its long‐standing use; however, high centrifugal forces may induce vesicle aggregation and deformation and reduce biological activity, compromising both purity and functional relevance [14]. Thus, we hypothesized that (i) SEC would provide higher reproducibility and lower isolation‐driven bias than UC + SEC in equine blood EV preparations, and (ii) serum versus plasma would yield systematically different apparent EV cargo due to coagulation/platelet activation and matrix‐specific co‐isolates (e.g., lipoproteins and RNP complexes).

Standardizing blood‐derived EV isolation in the horse is critical to improve the quality and comparability of veterinary studies and to enable future translational work. The horse is a well‐established large‐animal model across key human‐relevant domains: asthma, which recapitulates airway remodeling and endotypes and enables two‐way translational insight [15, 16]; reproduction, spanning assisted reproductive technologies and reproductive aging/aneuploidy [17, 18]; exercise physiology, with convergent immune and cellular responses to strenuous effort [19]; and osteoarthritis (OA), providing a naturally occurring, tractable counterpart to human OA [20]. Additional parallels include metabolic syndrome/insulin dysregulation [21], atrial fibrillation—with spontaneous AF in horses [22, 23]—and tendon injury biology, where the superficial digital flexor tendon mirrors the human Achilles [24, 25]. These translational links underscore the added value of robust, reproducible EV protocols.

To address methodological gaps in the equine context, we compared EVs isolated from plasma and serum using SEC alone or combined UC + SEC, assessing morphology and concentration by TEM and NTA, and cargo composition by mass‐spectrometry‐based proteomics. Rather than asserting protocol superiority, our goal was to quantify how matrix (serum vs. plasma) and workflow (SEC vs. UC + SEC) influence EV yield, marker detection, and proteomic reproducibility in horses, and to determine whether the UC step provides added value or can be omitted without compromising quality.

## 2 Materials and Methods

### 2.1 Sample Collection

The inclusion criteria for the horses in this study were the absence of clinical symptoms and laboratory indicators of disease. Additional criteria required that the animals had not received drugs or vaccinations within the two weeks preceding sample collection. Animals were housed individually in well‐bedded stalls at a single training center with a unified and standardized feeding regimen. Peripheral blood samples were obtained in the morning from six healthy, adult horses in a fasted state. The animals were sampled before routine procedures were performed in healthy horses (e.g., castration or routine health examinations). Blood was collected atraumatically from the jugular vein using 21‐gauge needles into EDTA‐buffered vacuum tubes (for plasma collection) and plain tubes for serum (BD Vacutainer). As only peripheral blood was collected and no additional interventions were undertaken for research purposes, the work was classified as non‐experimental and did not require ethical approval, following European Directive 2010/63/EU and Polish legislation. The initial 4 mL of blood was used for routine biochemical analyses, after which additional samples were collected for plasma and serum processing. Because anticoagulant choice can substantially alter EV signatures, consistent use of a single, well‐justified anticoagulant is essential in biomarker discovery studies [26]. EDTA was therefore selected as the most commonly used and clinically standardized anticoagulant in plasma‐based EV research, ensuring reproducibility, cross‐study comparability, and future translational applicability of the proposed workflow.

**FIGURE 1:**
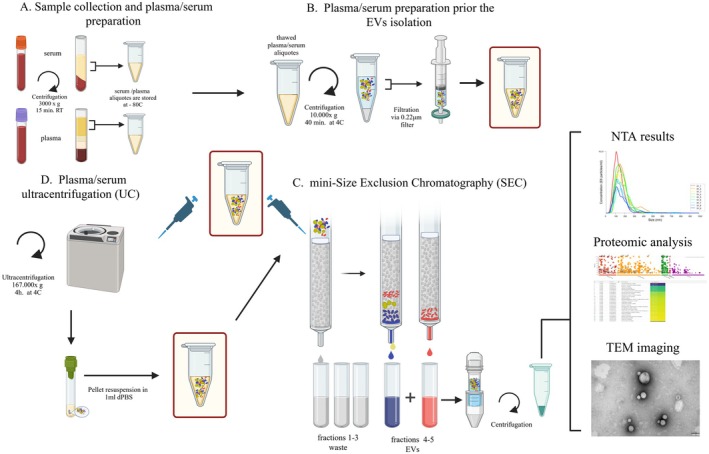
Workflow for plasma/serum extracellular vesicle (EV) isolation and characterization. (A) Plasma/serum collection and initial processing by centrifugation. Aliquots are stored at −80°C. (B) Plasma/serum preparation before EV isolation: centrifugation (10 000× *g*, 40 min, 4°C) and filtration through a 0.22 μm filter. (C) EV isolation using mini‐size exclusion chromatography (SEC): fractions 1–3 are discarded, fractions 4–5 contain EVs and are concentrated by centrifugation. (D) Alternative method: EV isolation by ultracentrifugation (167 000× *g*, 4 h, 4°C) and pellet resuspension in dPBS. Isolated EVs are subjected to nanoparticle tracking analysis (NTA), proteomic analysis, and transmission electron microscopy (TEM) imaging. Image created by Biorender.com.

Following collection, samples were maintained in an upright position at 4°C and processed within one hour. Platelet‐free plasma (PFP) and serum were prepared by centrifugation at 3000× *g* for 15 min at room temperature (RT). Subsequently, the upper layer of the supernatant (approximately 1500 μL) was carefully aspirated to obtain PFP and serum, which were immediately frozen and stored at −80°C for a maximum of one month before analysis (Figure 1A).

Prior to analysis, frozen plasma and serum samples were thawed and subjected to a second centrifugation at 10 000× *g* for 40 min at 4°C (MPV 260R, and fixed‐angle rotor REF11199) to remove residual cells, larger vesicles, and remaining debris. Next, the supernatant was carefully transferred to a sterile 1.5‐mL microcentrifuge tube and then filtered using a 0.22 μm filter (MILLEX GP filter Unit). The plasma/serum thus prepared was subsequently subjected to further processing (Figure 1B).

### 2.2 EVs Separation From Plasma and Serum Using a Homemade Mini‐SEC Protocol

The homemade mini‐SEC columns (10 mL) (Econo‐Pac Chromatography Columns, Biorad) were prepared as described by Ludwig et al. [27, 28] using Sepharose CL‐2B (Cytiva, Merck) suspension. Columns were gently flushed with dPBS and then filled with dPBS with preservative (0.05% sodium azide) and stored at 4°C. EVs isolation was performed once the columns reached RT and had been rinsed twice with dPBS. The UC + SEC workflow was included as a commonly used two‐step enrichment strategy to test whether an ultracentrifugation pre‐enrichment step improves sample purity and proteomic reproducibility compared with SEC alone in equine serum/plasma [29]. A 1 mL precleared and filtered thawed plasma or serum sample was placed in the column to elute fraction 1. When it stopped dripping, 1 mL of dPBS was added to elute fraction 2. The procedure was repeated. Fractions 1–3 (each 1 mL) were a “waste fraction”, whereas fractions 4 and 5 (each 1 mL) were enriched in small EVs (sEVs). Between consecutive isolations, the columns were rinsed twice with dPBS. Columns were reused up to twice. To verify column stability across re‐uses, we tracked protein recovery, total LC–MS/MS protein identifications, and consistent detection of EV markers (CD9, CD63, PDCD6IP) between the first and second use of each SEC column. Next, the fractions, 4 and 5, were pooled (Amicon Ultra 2 mL Centrifugal Filters, MERCK) and concentrated by centrifugation (MPW‐351R centrifuge, with swing‐out rotor REF 12436B) at 3900× *g* for 30 min at RT, and stored in 50 μL at −80°C for further processing (Figure 1C).

### 2.3 EVs Separation From Plasma and Serum Using Differential Ultracentrifugation and Homemade Mini‐SEC

A 1 mL precleared and filtered thawed plasma or serum sample was processed according to a modified Zhang et al. protocol [30, 31]. The sample was transferred to a centrifuge tube (Thermo Scientific, Tube PC THICKWALL, 03‐237) and centrifuged at 167 000× *g* for 4 h at 4°C in Sorvall MX120+ micro‐ultracentrifuge (Thermo Scientific; fix‐angle rotor S58‐A 2612; acceleration 9 and deceleration 7). Next, the pellet was resuspended in 1 mL dPBS and transferred into homemade mini‐SEC columns (Figure 1D).

### 2.4 EV Characterization

#### 2.4.1 Sample Preparation and TEM Imaging

A 10 μL aliquot of the vesicle suspension was applied onto a 400‐mesh copper TEM grid. After 1 min of incubation, the excess liquid was removed using filter paper, and 10 μL of 1% aqueous uranyl acetate solution was immediately applied for negative staining. After 40 s, the stain was blotted off with filter paper, and the grid was left to air‐dry. The samples were imaged using a JEM‐1400 transmission electron microscope (JEOL Co., Japan) operated at 80 kV and equipped with an 11‐megapixel MORADA G2 camera (EMSIS GmbH, Germany).

#### 2.4.2 Nanoparticle Tracking Analysis (NTA)

The analyzed samples consisted of isolates of equine serum‐derived EVs. The parameters assessed included the mean size of particles/aggregates and the particle/aggregate size distribution in liquid suspensions. The measurement range spanned from 20 to 450 nm. Particle characterization was performed using Nanoparticle Tracking Analysis (NTA), which enables the detection and analysis of nanoparticle motion trajectories. Measurements were conducted using a NanoSight NS500 instrument (NanoSight Ltd., serial number: 80336/A) equipped with a 405 nm blue diode laser. The system was operated using NTA software version 2.3 (build 0025, NanoSight Ltd.). All analyses were performed according to the ASTM E2834‐12 standard. Samples were received frozen, thawed immediately before analysis, and diluted 1:1 with PBS. Measurements were performed in triplicate sets (nine videos total), and results are reported as batch averages. Minor vibration during analysis was detected, but did not prevent data acquisition. Following the proteomic analysis, Serum‐SEC was selected for TEM and NTA as it provided the most reproducible and EV‐enriched preparation, allowing confirmatory structural and size characterization to be performed on the workflow identified as optimal in this study.

### 2.5 Proteomic Analysis

#### 2.5.1 Sample Preparation

Extracellular vesicles (EVs), suspended in 100 μL of PBS, were lysed by adding SDS to a final concentration of 1%. The samples were then vortexed for 15 min, heated at 95°C for 10 min, and sonicated for 15 min in an ultrasonic bath. To reduce disulfide bonds, samples were incubated for 60 min with 10 mM tris(2‐carboxyethyl)phosphine, followed by alkylation with 30 mM methyl methanethiosulfonate (MMTS) for 5 min at room temperature. Protein digestion was performed using a modified SP3 protocol [32]. A bead slurry was prepared by mixing equal volumes of hydrophilic and hydrophobic Sera‐Mag Carboxyl magnetic particles (Cytiva, cat. no. 65152105050250 and 45152105050250), washing them three times with MS‐grade water, and resuspending to a final concentration of 10 μg/μL. Forty microliters of the bead slurry was added to the samples and vortexed at 1000 rpm for 15 min. Next, 900 μL of 0.1% formic acid (FA) in acetonitrile (Evosep solvent B) was added, and samples were vortexed again for 20 min, briefly centrifuged, and placed on a magnetic rack for 15 min to collect the beads. The supernatants were discarded. Beads were washed three times with 1 mL of 80% ethanol and twice with 1 mL of acetonitrile. After drying the beads for 15 min at 95°C, proteins were digested overnight at 37°C with 2 μg of trypsin (Promega) in 100 μL of 100 mM ammonium bicarbonate buffer under constant shaking. The resulting peptide mixtures were transferred to fresh tubes, and the beads were washed twice with 50 μL of water. Combined eluates were acidified with 0.1% FA.

To minimize batch effects, sample order was randomized for SEC/UC and SEC alone processing and for Liquid Chromatography–Tandem Mass Spectrometry (LC–MS/MS) injections.

#### 2.5.2 Mass Spectrometry

Peptide samples were analyzed on an integrated LC–MS system consisting of an Evosep One chromatograph (Evosep Biosystems, Odense, Denmark) coupled to an Orbitrap Exploris 480 mass spectrometer (Thermo Fisher Scientific, Bremen, Germany). From each sample, 100 μL of the digest (equivalent to 50% of the total volume) was loaded onto disposable Evotips C18 columns, following the manufacturer's protocol. The bound peptides were washed twice with 150 μL of solvent A (0.1% FA in water) and covered with 300 μL of the same solvent. Separation was carried out using the 88‐min predefined gradient (15 samples/day mode) at 220 nL/min on a C18 analytical column (EV1106, Dr. Maisch C18 AQ, 1.9 μm, 150 μm ID × 15 cm). Data acquisition was performed in positive ion mode using data‐dependent acquisition (DDA). MS1 spectra were collected at 60000 resolution with a normalized AGC target of 300%, an automatic maximum injection time, and a scan range of 300–1600 m/z. Up to 40 most abundant precursor ions were selected for MS2 fragmentation using a 1.6 m/z isolation window. MS2 spectra were acquired at a resolution of 15 000, with standard AGC settings and automatic injection time. A dynamic exclusion window of 20 s was applied with a precursor mass tolerance of ±10 ppm and a minimum signal intensity of 5 × 10^3^. Fragmentation was performed using higher‐energy collisional dissociation with a normalized collision energy of 30%. The spray voltage was set to 2.1 kV, funnel RF level to 40, and capillary temperature to 275°C.

#### 2.5.3 Data Analysis

Raw data were processed using Proteome Discoverer (version 3.2, Thermo Fisher Scientific). Spectral recalibration was performed using the Spectrum Files RC node, followed by data filtering and pre‐processing with the Spectrum Selector module before database searching. Peptide identification was performed using the Sequest HT search engine against the *Equus caballus* reference proteome (version 202504, one entry per gene), allowing up to two missed cleavages. Methylthio (C) was set as a fixed modification, and oxidation (M) as a variable one. Identified peptide‐spectrum matches (PSMs) were evaluated using Percolator. A concatenated target‐decoy approach was used to estimate false discovery rates, applying strict and relaxed FDR thresholds of 1% and 5%, respectively. Peptides were assembled into protein groups using the Protein Scorer and Protein Grouping nodes, and validated with the Peptide Validator and PSM Grouper modules. Identifications with at least medium confidence were retained for downstream analysis.

#### 2.5.4 Statistical and Reproducibility Strategy

Because this study was designed as a methodological comparison (*n* = 6) focused on analytical reproducibility and workflow‐dependent biases, we primarily used descriptive and overlap‐based metrics rather than inferential testing of protein abundance. Protein identifications were filtered at strict protein‐level FDR (< 1%) for high‐confidence comparisons, as described above. Reproducibility was summarized as (i) the number of proteins detected in ≥ 3/6 biological replicates and (ii) the “core” proteome detected in all 6/6 replicates; canonical EV markers were likewise interpreted using a ≥ 3/6 consistency threshold. Group‐level results are reported using counts and overlaps to reduce sensitivity to non‐random missingness typical of DDA proteomics. Functional enrichment analyses were corrected for multiple testing using g:SCS and interpreted at FDR < 0.05.

#### 2.5.5 Functional Enrichment Analysis of Unique Proteins

High‐confidence protein lists (protein *q*‐value FDR < 1%) were generated for each EV preparation (Serum_SEC, Plasma_SEC) and compared to identify proteins unique to each group. Unique protein sets were analyzed in g:Profiler (https://biit.cs.ut.ee/gprofiler/gost, organism: *Homo sapiens* ) using GO:BP, GO:MF, GO:CC, KEGG, and WikiPathways databases. The significance threshold was set to FDR < 0.05 with g:SCS multiple testing correction.

The top 15 enriched terms per group were extracted based on adjusted *p*‐values and compared to identify unique and shared categories. Visualizations (bar plots of –log_10_(FDR)) were created in Python using pandas and matplotlib.

#### 2.5.6 Comparative Analysis of Identified EV Proteins

Comparative analysis of identified EV proteins was performed using FunRich v3.1.3. For each group, proteins consistently detected in at least 3 of 6 biological replicates were selected. Equine proteins were mapped to human gene symbols based on sequence homology and compared with the Vesiclepedia reference dataset (top 1000 proteins) (http://www.microvesicles.org/) and with plasma EV proteins reported in the meta‐analysis by Vallejo et al. [33]. Additionally, overlaps were assessed between EV isolation methods (SEC vs. UC + SEC) and sources (plasma vs. serum).

## 3 Results

### 3.1 Proteomic Profiling

Across individual samples (IDs per LC–MS/MS run), serum‐derived EV preparations yielded higher typical identifications than plasma. Median IDs (range) were: Serum_SEC 446 (270–663), Serum_UC + SEC 351 (110–722), Plasma_SEC 358.5 (315–1258), and Plasma_UC + SEC 312 (273–338). Inter‐sample dispersion was widest for Serum_UC + SEC and Plasma_SEC, the latter driven by one sample with markedly elevated IDs (S3 = 1258) (Table 1).

**TABLE 1:** Summary of protein identifications (IDs) and the presence of selected exosomal markers (tetraspanins CD9, CD63, CD81, CD82, TSPAN9, TSPAN7, TSG101, and PDCD6IP) in extracellular vesicles isolated from plasma and serum using size‐exclusion chromatography (SEC) or ultracentrifugation combined with SEC (UC + SEC).

Group	Sample no	IDs	Tetraspanin CD9	Tetraspanin CD63	Tetraspanin CD81	Tetraspanin CD82	Tetraspanin TSPAN9	Tetraspanin TSPAN7	TSG101	PDCD6IP
Plasma_SEC	S1	368	Yes	No	Yes	No	No	No	No	Yes
Plasma_SEC	S2	351	No	No	Yes	No	No	No	No	Yes
Plasma_SEC	S3	1258	No	No	Yes	No	Yes	No	Yes	Yes
Plasma_SEC	S4	366	Yes	No	Yes	No	No	Yes	No	Yes
Plasma_SEC	S5	315	Yes	No	No	No	No	No	No	Yes
Plasma_SEC	S6	327	Yes	No	Yes	No	No	No	No	Yes
Plasma_Ultra‐SEC	S1	338	Yes	No	Yes	No	No	No	No	Yes
Plasma_Ultra‐SEC	S2	296	No	No	Yes	No	No	No	No	Yes
Plasma_Ultra‐SEC	S3	337	No	No	No	No	No	No	No	Yes
Plasma_Ultra‐SEC	S4	291	No	No	No	No	No	No	No	No
Plasma_Ultra‐SEC	S5	328	Yes	No	No	No	No	No	No	Yes
Plasma_Ultra‐SEC	S6	273	No	No	No	Yes	No	No	No	No
Serum_SEC	S1	663	Yes	Yes	No	No	Yes	Yes	No	Yes
Serum_SEC	S2	448	Yes	Yes	No	No	Yes	Yes	No	Yes
Serum_SEC	S3	585	Yes	Yes	No	No	Yes	Yes	No	Yes
Serum_SEC	S4	444	Yes	Yes	Yes	No	Yes	Yes	No	Yes
Serum_SEC	S5	270	No	Yes	No	No	No	Yes	No	No
Serum_SEC	S6	346	Yes	Yes	No	No	Yes	Yes	No	Yes
Serum_Ultra‐SEC	S1	722	Yes	Yes	Yes	No	Yes	Yes	Yes	Yes
Serum_Ultra‐SEC	S2	342	Yes	Yes	No	No	Yes	Yes	No	Yes
Serum_Ultra‐SEC	S3	531	Yes	Yes	No	No	Yes	Yes	No	Yes
Serum_Ultra‐SEC	S4	110	No	No	No	No	No	Yes	No	No
Serum_Ultra‐SEC	S5	193	Yes	Yes	No	No	No	No	No	No
Serum_Ultra‐SEC	S6	360	Yes	Yes	No	Yes	Yes	Yes	No	Yes

*Note:* Each row represents an individual sample (S1–S6) within the corresponding group.

At the group level, the total number of proteins identified in at least one sample was highest for Plasma_SEC (1423), followed by Serum_SEC (877), Serum_UC + SEC (874), and Plasma_UC + SEC (591). Across replicates, reproducibility favored Serum_SEC: proteins detected in ≥ 3/6 samples numbered 464 for Serum_SEC versus 366 for Plasma_SEC, and the ‘core’ proteome shared by all six replicates was likewise larger for Serum_SEC (191 vs. 185), confirming higher cross‐sample consistency.

Canonical EV markers exhibited distinct detection patterns depending on the biofluid and isolation method (threshold: detected in ≥ 3/6 samples). In Plasma_SEC, CD9, CD81, and PDCD6IP were consistently detected, whereas CD63, CD82, TSPAN9, TSPAN7, and TSG101 did not meet this criterion. In Plasma_UC + SEC, only PDCD6IP was detected, with all tetraspanins and TSG101 not being identified. In Serum_SEC, CD9, CD63, TSPAN9, TSPAN7, and PDCD6IP were consistently detected, while CD81, CD82, and TSG101 did not meet the threshold. A similar pattern was observed in Serum_UC + SEC, with consistent detection of CD9, CD63, TSPAN9, TSPAN7, and PDCD6IP, and no consistent detection of CD81, CD82, or TSG101 (Table 2).

**TABLE 2:** Number of proteins identified in extracellular vesicles (EVs) isolated from plasma and serum using size‐exclusion chromatography (SEC) or ultracentrifugation combined with SEC (UC + SEC).

Group	Identified in at least 1 sample	Identified in at least 3 samples	Identified in all 6 samples	Tetraspanin CD9	Tetraspanin CD63	Tetraspanin CD81	Tetraspanin CD82	Tetraspanin TSPAN9	Tetraspanin TSPAN7	TSG101	PDCD6IP
Plasma_SEC	1423	366	185	Yes	No	Yes	No	No	No	No	Yes
Plasma_Ultra‐SEC	591	304	137	No	No	No	No	No	No	No	Yes
Serum_SEC	877	464	191	Yes	Yes	No	No	Yes	Yes	No	Yes
Serum_Ultra‐SEC	874	378	78	Yes	Yes	No	No	Yes	Yes	No	Yes

*Note:* Values are shown for proteins identified in at least one sample, in at least three samples, and in all six samples per group. The presence of canonical EV markers (tetraspanins CD9, CD63, CD81, CD82, TSPAN9, TSPAN7, TSG101, and PDCD6IP) detected in at least three samples is also indicated.

**FIGURE 2:**
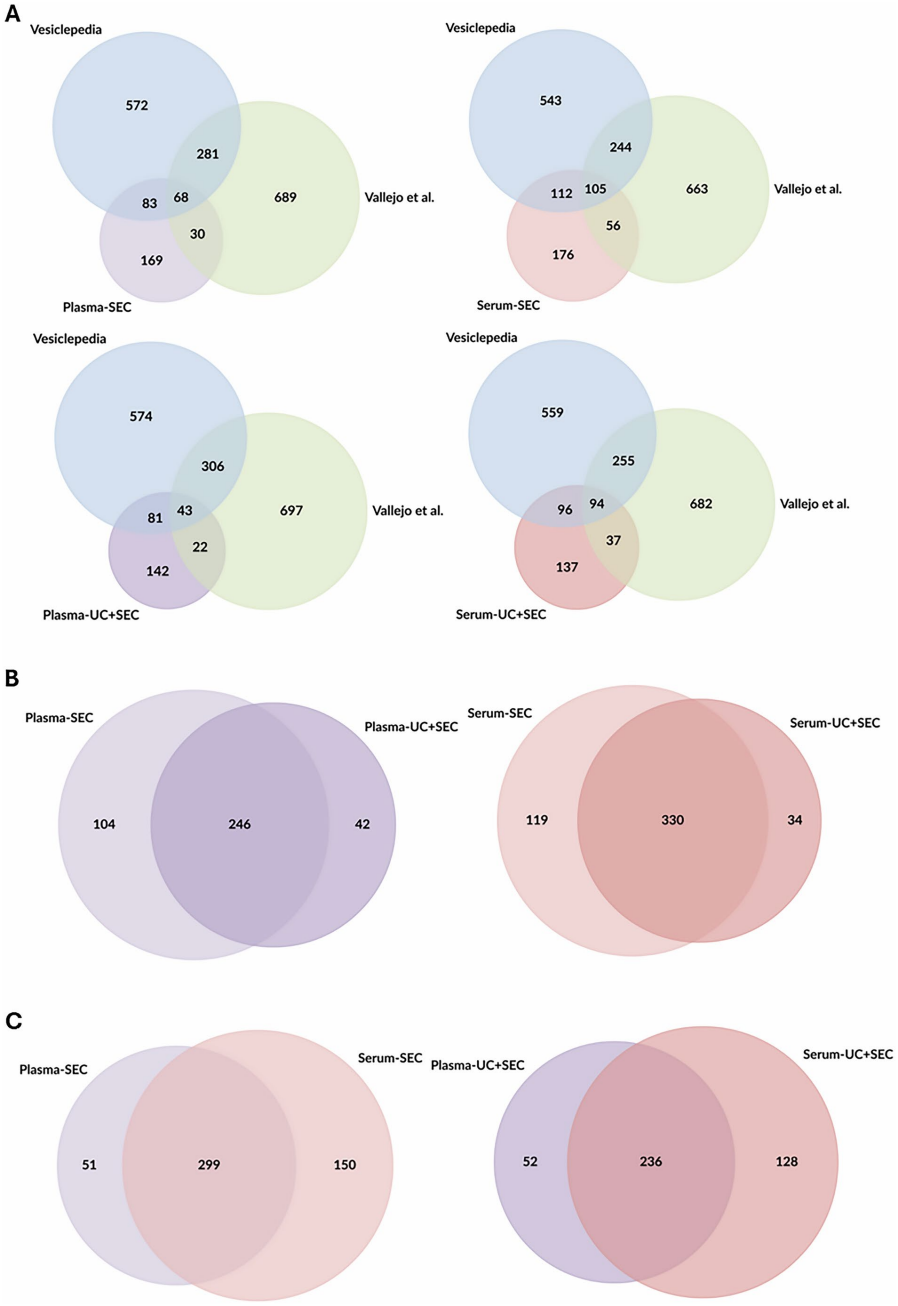
Venn diagrams showing the overlap of identified proteins between Vesiclepedia, Vallejo et al., and EV protein datasets obtained from plasma and serum using size‐exclusion chromatography (SEC) and combined ultracentrifugation plus SEC (UC + SEC). (A) Comparison of Vesiclepedia, Vallejo et al., and plasma/serum EV datasets. (B) Overlap of proteins identified in plasma (SEC vs. UC + SEC) and serum (SEC vs. UC + SEC). (C) Direct comparison of plasma and serum EV proteomes isolated by different methods.

Venn diagrams showing the overlap of identified proteins between Vesiclepedia, Vallejo et al., and EV protein datasets obtained from plasma and serum using size‐exclusion chromatography (SEC) and combined ultracentrifugation plus SEC (UC + SEC) (Figure 2). Complete proteomics results for EV‐enriched fractions from equine plasma and serum processed with SEC/Ultra‐SEC have been presented in Table S1.

### 3.2 Functional Differences Between Serum_SEC and Plasma_SEC

We compared high‐confidence proteins identified in EVs from serum (Serum_SEC) and plasma (Plasma_SEC). Across groups, 827 (Serum_SEC) and 1276 (Plasma_SEC) (high‐confidence (FDR < 1%)) proteins were detected, with 644 shared, 183 unique to Serum_SEC, and 632 unique to Plasma_SEC.

**FIGURE 3:**
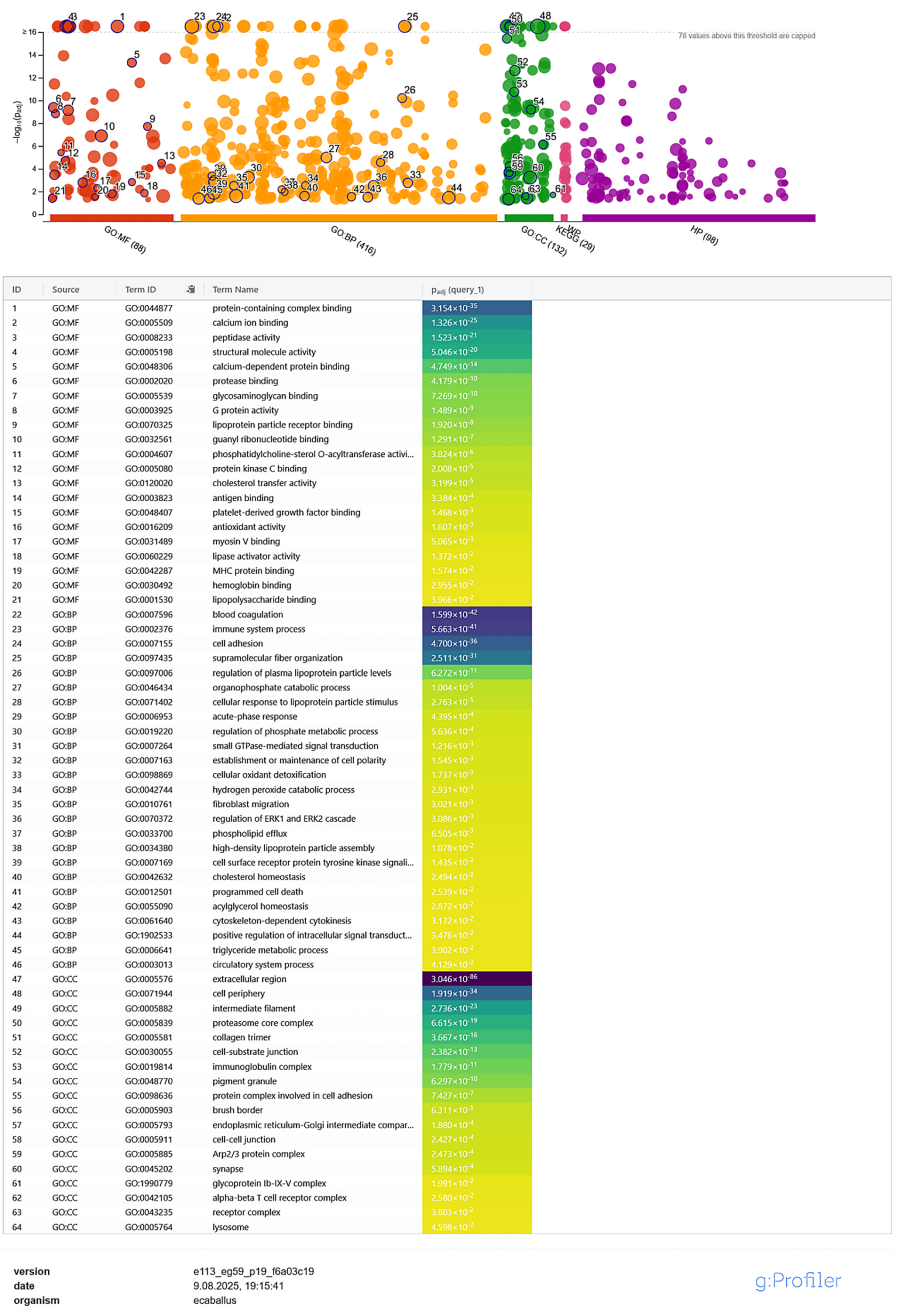
Functional enrichment analysis of unique protein sets from Serum_SEC extracellular vesicles. Volcano‐style Manhattan plots (top) and top enriched terms (bottom) generated in g:Profiler ( *Equus caballus* , GO:BP/MF/CC, KEGG, WikiPathways, g:SCS correction, FDR < 0.05). Bars indicate –log_10_(FDR) values; representative top terms are listed in the heatmap table.

Functional enrichment analysis performed with g:Profiler identified multiple significantly overrepresented Gene Ontology (GO) terms in the “high confidence” protein sets for both Serum_SEC and Plasma_SEC. In Serum_SEC (Figure 3), the most strongly enriched GO:MF terms included *structural molecule activity* (GO:0005198, adj. *p* = 1.32 × 10^−47^), *protein‐containing complex binding* (GO:0044877, 1.05 × 10^−37^), and *hydrolase activity* (GO:0016787, 1.35 × 10^−17^). Among GO:BP, processes such as *protein metabolic process* (GO:0019538, 1.35 × 10^−7^), *complement activation* (GO:0006956, 1.33 × 10^−27^), and *blood coagulation* (GO:0007596, 7.08 × 10^−23^) were prominent. GO:CC terms with the highest significance included *extracellular space* (GO:0005615, 5.84 × 10^−15^) and *cornified envelope* (GO:0001533, 2.38 × 10^−15^). Unless stated otherwise, functional enrichment refers to unique protein**s** per group.

**FIGURE 4:**
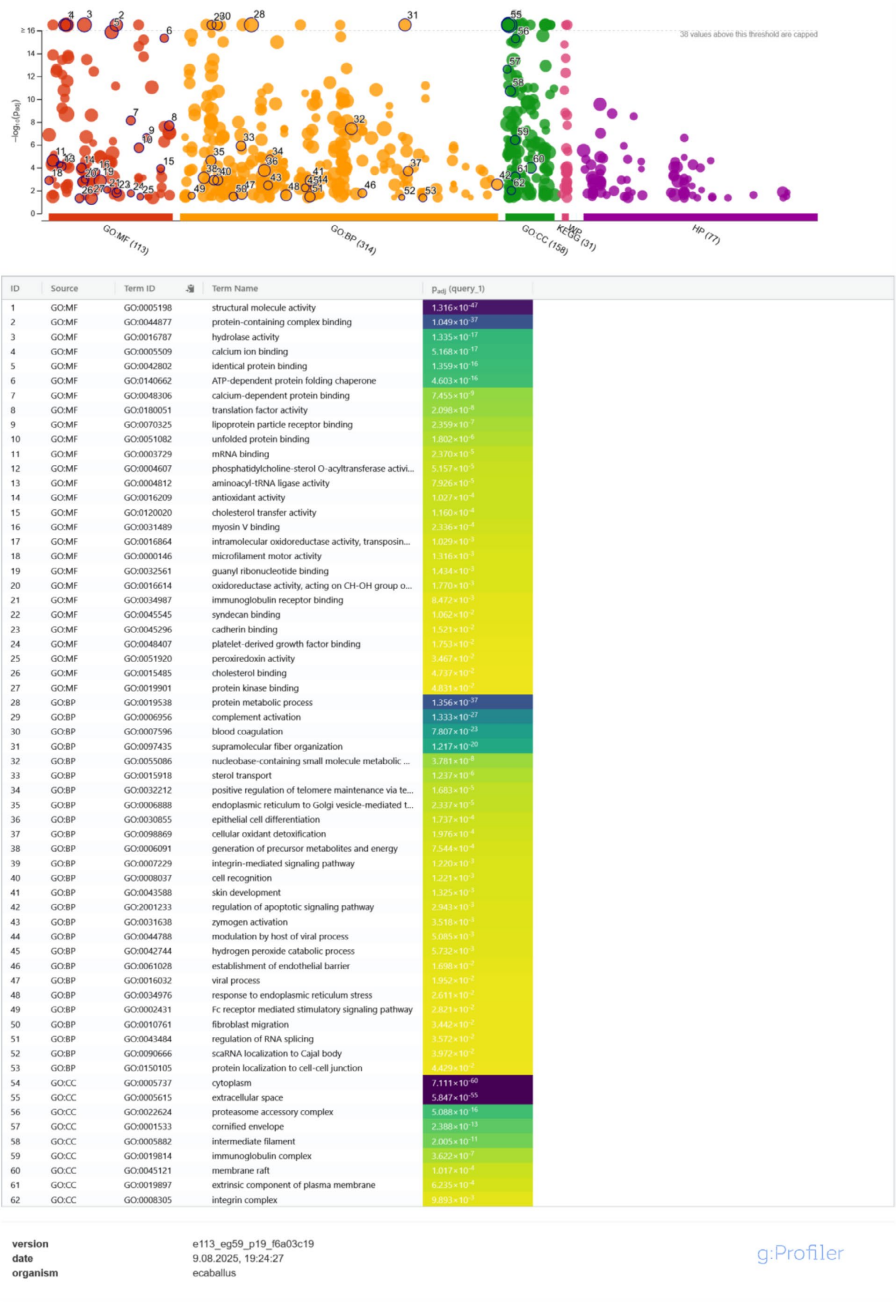
Functional enrichment analysis of unique protein sets from Plasma_SEC extracellular vesicles. Manhattan plots (top) and heatmaps of the top enriched terms (bottom) generated in g:Profiler ( *Equus caballus* , GO:BP/MF/CC, KEGG, WikiPathways, g:SCS correction, FDR < 0.05). Bars represent –log_10_(FDR) values; representative top terms are listed in the heatmap table.

In Plasma_SEC (Figure 4), enrichment was dominated by binding‐related GO:MF terms, such as *protein‐containing complex binding* (GO:0044877, 3.15 × 10^−35^), *calcium ion binding* (GO:0005509, 1.32 × 10^−30^) and *peptidase activity* (GO:0008233, 5.24 × 10^−28^). For GO:BP, notable processes included *immune system process* (GO:0002376, 5.65 × 10^−41^), *blood coagulation* (GO:0007596, 1.59 × 10^−42^), and *plasma lipoprotein particle levels* (GO:0097009, 6.27 × 10^−13^). Among GO:CC, terms such as *extracellular region* (GO:0005576, 3.04 × 10^−36^) and *cell periphery* (GO:0071944, 1.19 × 10^−34^) were highly significant.

Enrichment analyses (GO:BP/MF/CC, KEGG, WikiPathways) were run on unique sets (Table 3). Plasma_SEC unique proteins were enriched for translational and proteostasis‐related terms (e.g., structural constituent of ribosome, RNA binding, ATP‐dependent chaperone activity), while Serum_SEC unique proteins were enriched for immune/adhesion/cytoskeletal and hemostasis‐related terms (e.g., immune system process, cell adhesion, supramolecular fiber organization, blood coagulation) (Figure S1).

**TABLE 3:** Top enriched terms for unique proteins.

Source	Term name	Term ID	FDR
*(A) Serum_SEC unique—TOP 10*			
GO:CC	Cell periphery	GO:0071944	1.10e−11
GO:CC	Plasma membrane	GO:0005886	2.39e−10
GO:BP	Immune system process	GO:0002376	1.61e−09
GO:CC	Vesicle	GO:0031982	2.62e−08
GO:BP	Cell activation	GO:0001775	8.52e−08
GO:CC	Cytoplasmic vesicle	GO:0031410	1.59e−07
GO:CC	Intracellular vesicle	GO:0097708	1.87e−07
GO:BP	Cell adhesion	GO:0007155	2.15e−07
GO:BP	Supramolecular fiber organization	GO:0097435	2.44e−07
GO:CC	Cell surface	GO:0009986	4.76e−07
*(B) Plasma_SEC unique—TOP 10*			
GO:CC	Cytoplasm	GO:0005737	1.87e−72
GO:CC	Cytosol	GO:0005829	7.90e−57
GO:CC	Cytosolic ribosome	GO:0022626	2.00e−54
GO:BP	Translation	GO:0006412	1.25e−44
GO:CC	Intracellular anatomical structure	GO:0005622	1.00e−40
GO:CC	Ribosomal subunit	GO:0044391	2.00e−40
GO:CC	Ribosome	GO:0005840	2.71e−35
GO:CC	Protein‐containing complex	GO:0032991	2.49e−32
GO:MF	Structural constituent of ribosome	GO:0003735	3.83e−32
GO:BP	Protein metabolic process	GO:0019538	8.10e−30

### 3.3 TEM and NTA Confirmation

**FIGURE 5:**
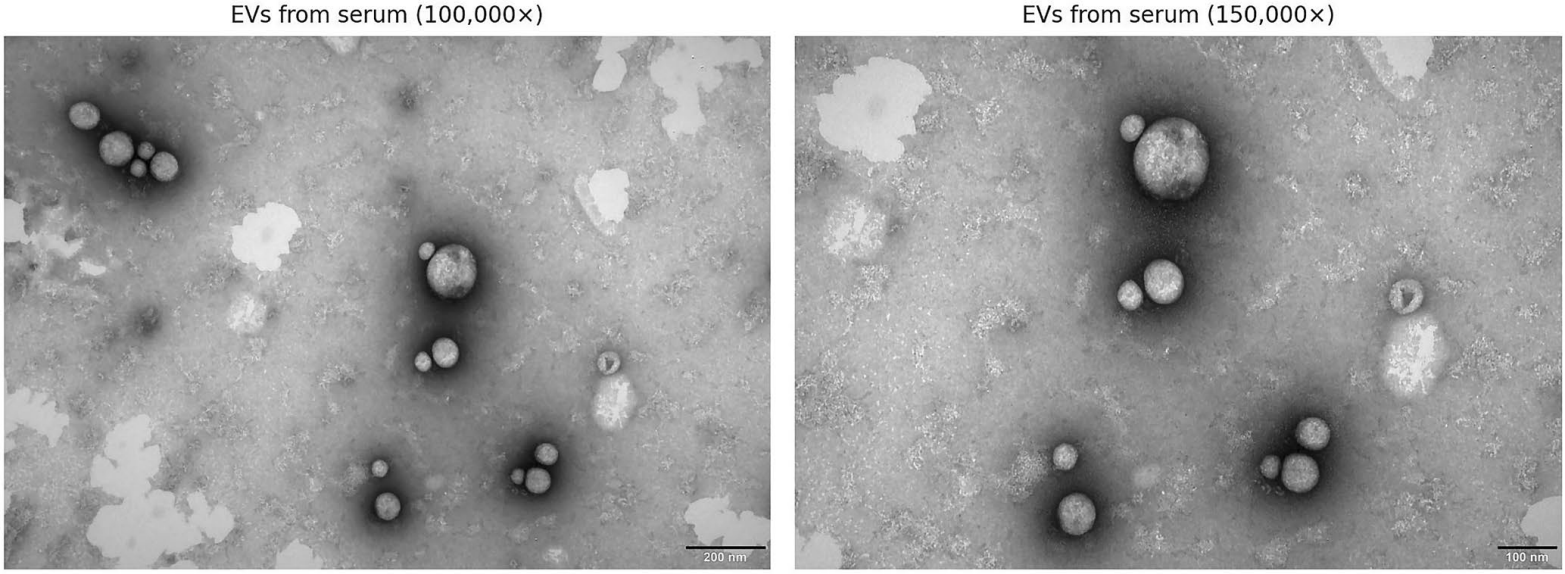
Transmission electron microscopy (TEM) images of extracellular vesicles (EVs) isolated from serum using size exclusion chromatography (SEC). The vesicles exhibit typical round, cup‐shaped morphology. Left: magnification 100 000×. Right: magnification 150 000 ×.

Following the proteomic analysis, samples isolated from serum using the more optimal SEC method (Serum_SEC) were selected for further characterization by TEM and NTA. TEM confirmed the presence of typical round, cup‐shaped vesicles in all sample types and isolation methods, with sizes ranging from 50 to 150 nm (Figure 5).

**FIGURE 6:**
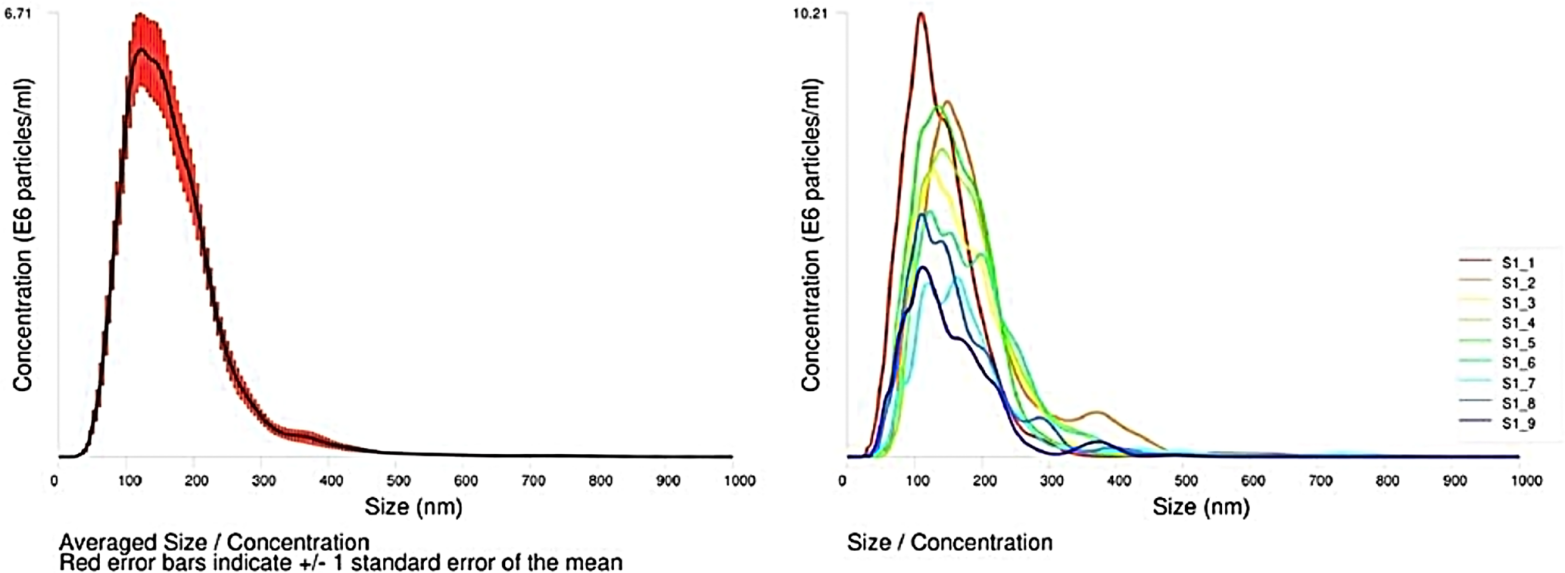
Characterization of extracellular vesicles (EVs) isolated from serum using size exclusion chromatography (SEC). Nanoparticle Tracking Analysis (NTA) shows the averaged size distribution with a predominant population in the 100–200 nm range, consistent with small EVs, along with individual sample traces.

NTA measurements were performed using a NanoSight NS500 (405 nm laser) with PBS as dispersant. The vesicle preparation displayed a mean particle diameter of 170.0 ± 5.9 nm and a modal diameter of 129.0 ± 6.2 nm, with a standard deviation of 80.0 ± 5.6 nm, indicating a heterogeneous size distribution. The particle concentration was (9.11 ± 0.78) × 10^8^ particles/mL, based on a total of 10 512 completed tracks (Figure 6). The size distribution profile showed a predominant population in the 100–200 nm range, consistent with small extracellular vesicles, with a tail towards larger particles. The average drift velocity during analysis was 547 nm/s.

## 4 Discussion

To advance understanding of EVs in equine veterinary medicine, we compared vesicles isolated from equine plasma and serum using SEC and differential UC + SEC. Adding UC prior to SEC did not improve marker consistency but increased between‐sample variability and reduced the shared (“core”) proteome, indicating that UC may be unnecessary or even detrimental for reproducible blood‐EV proteomics in this equine setting. Although UC − SEC is commonly used in biofluid EV studies [28, 31, 34], our data show its benefit is matrix‐ and species‐dependent and that, in equine serum/plasma, the added UC step did not improve analytical performance. Serum processed with SEC yielded the most reproducible EV proteomes—both by the largest ≥ 3‐of‐6 and all‐6 overlaps. The most consistent detection of key EV markers—whereas plasma, particularly with UC + SEC, showed lower marker consistency and, for Plasma_SEC, greater between‐sample variability in identifications. Overall, SEC was a simpler and more efficient method, producing more identified proteins with better replicate consistency, while UC, though long established, was more labor‐intensive, gave lower protein recovery, and introduced greater variability [21, 22, 23, 24, 25]. Our data thus confirm SEC as an effective, reliable approach for EV isolation from equine serum and plasma, in line with recent reports [26, 40]. In group‐level metrics, Serum_SEC showed a reproducibility advantage over Plasma_SEC, with more proteins detected in ≥ 3/6 replicates (464 vs. 366) despite Plasma_SEC having more “any‐sample” identifications, as all reproducibility values (≥ 3/6 and all‐6) refer to high‐confidence protein IDs (protein‐level FDR < 1%), explaining numerical differences from ‘any‐sample’ totals.

### 4.1 Methodological Context and Species‐Specific Considerations

No current methodology can isolate the entire EV population—or exclusively EVs—from plasma, serum, or other body fluids because of size/density overlap among EVs, platelets, and lipoproteins. Co‐isolation of lipoproteins is unavoidable, and their concentrations may exceed EVs by up to a million‐fold [26]. In equine blood, HDL predominance (> 60% of total lipoproteins) is a major species‐specific constraint that can increase lipoprotein carry‐over into EV preparations [11, 12]. Although SEC provides gentler, size‐based partitioning, complete exclusion of HDL remains challenging in HDL‐rich matrices, particularly when lipoproteins and EVs form complexes and/or acquire a protein corona that can affect apparent size and co‐elution. Consistent with this, Plasma_SEC showed enrichment of the GO term “plasma lipoprotein particle levels” (GO:0097009), suggesting residual lipoprotein‐associated cargo in the isolates, which is consistent with other studies [41]. Therefore, while SEC improved reproducibility compared with UC + SEC, we emphasize that additional mitigation steps (e.g., density‐based lipoprotein depletion prior to SEC) and routine monitoring of apolipoprotein markers (ApoA1/ApoB) should be considered when the goal is high‐specificity EV omics in horses. In this context, SEC's size‐based partitioning and gentle handling help reduce lipoprotein carry‐over while preserving vesicle integrity, which is advantageous for proteomics and RNomics [42, 43]. By contrast, UC readily co‐isolates HDL due to overlapping biophysical properties [41], and repeated high‐g steps may compact pellets, promote aggregation, and impair downstream readouts [25, 35, 36, 37, 38].

Although SEC improved reproducibility and reduced high‐abundance contaminants compared with UC, our data indicate that HDL co‐isolation cannot be fully excluded. Because HDL particles and EVs share similar buoyant densities (~1.063–1.21 g/mL), they are often co‐isolated in conventional workflows [41], and the high abundance of lipoproteins in plasma makes separation by size alone challenging [39, 41, 44]. Density‐based lipoprotein depletion before SEC has been shown to markedly reduce HDL contamination and improve detection of genuinely vesicular proteins. These species‐specific constraints argue for SEC‐based workflows as a pragmatic default in horses [44, 45], provided pre‐analytical variables are tightly controlled and reporting follows ISEV/ISMVs guidelines.

### 4.2 Choice of Blood‐Derived Material (Serum vs. Plasma)

Across both matrices, SEC yielded more identifications per run and better replicate consistency than UC + SEC. Although Plasma_SEC had the largest group‐level ‘any‐sample’ tally, serum (particularly with SEC) produced more consistent and cleaner EV populations in our hands. Coagulation‐related proteins present in plasma EV isolates can obscure biological signals and reduce specificity of proteomics; conversely, serum EVs can be enriched for platelet‐derived vesicles generated during clotting [8, 9, 46, 47]. While Serum_SEC provided the most reproducible proteomes overall, the functional tilt of the cargo differed by matrix: plasma skewed towards translation/proteostasis, whereas serum skewed towards immune/adhesion/hemostasis pathways.

Matrix‐dependent differences between serum‐ and plasma‐derived EV profiles are biologically plausible and likely reflect coagulation‐driven platelet activation and matrix‐specific co‐isolates. Serum formation can trigger platelet activation and release of platelet‐derived EVs and can enrich vesicle fractions in adhesion/coagulation‐associated proteins. In horses, hemostasis and platelet biology show species‐specific features (e.g., atypical vWF responsiveness and distinct activation‐marker behavior), and equine platelets appear highly sensitive to mechanical and biochemical stimuli—factors that may amplify pre‐analytical release of platelet‐derived EVs during collection and processing [48].

In parallel, blood EVs can adsorb soluble proteins (including coagulation and complement components) to form a “protein corona”, which may reshape the apparent EV proteome and influence downstream biological interpretation and uptake; plasma and serum can differ in the extent and composition of such extravesicular associations [47]. Finally, anticoagulated plasma may also retain non‐vesicular ribonucleoprotein complexes that can co‐isolate with EV fractions and contribute to translation/proteostasis‐related signatures, whereas clot formation may sequester or reduce carry‐over of these components into serum fractions. These mechanisms together provide a coherent biological framework for the observed serum–plasma divergence and reinforce the need for strict pre‐analytical standardization and transparent reporting in equine blood‐EV studies. Thus, our workflow follows a two‐stage biomarker development strategy in blood EV proteomics, in which EV enrichment serves as a biologically relevant pre‐fractionation step that mitigates the extreme dynamic range of plasma and improves detection of low‐abundance candidates during discovery [49]. Identified EV‐associated markers are subsequently translated to targeted quantification in whole plasma or serum after vesicle lysis, ensuring validation in a clinically relevant matrix that reflects the total (soluble and EV‐associated) protein pool and appropriate diagnostic performance metrics [50, 51].

The divergence from human EV literature (which often favors plasma) may be species‐driven: equine plasma strongly promotes erythrocyte aggregation via macromolecule‐mediated mechanisms (e.g., fibrinogen, globulins) [52], potentially promoting EV aggregation and reducing purification efficiency—thus tipping the balance towards serum for equine studies provided that processing is prompt and standardized. Finally, proteins and lipoproteins historically labeled as “contaminants” (e.g., albumin, ApoA1) can form a functional corona on EV surfaces, modulating EV properties [26, 53, 54]. Together with the HDL‐dominant lipoprotein profile of equine plasma, these species‐specific features complicate EV isolation and should be considered when selecting matrices and standardizing pre‐analytical protocols [12, 41].

### 4.3 Marker Detection Patterns and Interpretation

We assessed classical EV markers (CD9, CD63, CD81, CD82, TSPAN9, TSPAN7, TSG101, PDCD6IP). Tetraspanins are widely used as canonical EV markers [55, 56], yet CD9/CD63/CD81 are not uniformly expressed across sEV populations; their heterogeneous distribution mirrors donor‐cell phenotypes and likely extends to plasma, serum, and cerebrospinal fluid (CSF) in both humans and horses [57, 58]. In our dataset, CD63, TSPAN7, and TSPAN9 were consistently detected in serum, but rarely in plasma, aligning with evidence that CD63‐positive sEVs are enriched in human serum. CD9 levels were similar between matrices, consistent with contributions from platelet‐associated EVs [59]. Notably, TSG101 was inconsistent, suggesting caution when using TSG101 as a stand‐alone marker in equine samples [30, 60]. Moreover, phosphatidylserine‐positive EVs (medium/large EVs) can bind fibrinogen and accelerate fibrin polymerization [61, 62], indicating that both fibrinogen presence and clot formation shape EV profiles in plasma and serum—not only through EV generation but also via binding interactions that may bias isolation and detection.

Although we detected EV‐associated proteins such as CD9 and PDCD6IP, several widely used “canonical” markers (e.g., CD63, CD81, TSG101) were variably identified across matrices and workflows. This likely reflects a combination of biological and technical factors [56, 57, 58]. First, canonical EV markers are not universal: tetraspanin and ESCRT‐marker abundance varies substantially across EV subtypes and cell‐of‐origin, and blood EVs are expected to be enriched for platelet‐ and erythrocyte‐derived vesicles with a marker repertoire that may differ from commonly used human reference panels [47]. Second, species‐specific sequence variation and the still‐imperfect annotation of the *Equus caballus* proteome can reduce confident peptide assignment for some markers [55]. Third, discovery label‐free DDA proteomics on Orbitrap‐based MS is inherently biased against low‐abundance, highly hydrophobic multi‐pass membrane proteins (including tetraspanins), which often yield few MS‐friendly tryptic peptides and can be masked by the high dynamic range of plasma/serum background and co‐isolated nanoparticles. Accordingly, we interpret “low marker detection” as a limitation of marker universality and MS sensitivity rather than absence of EVs, and we emphasize the need for orthogonal validation and equine‐adapted marker panels in future studies.

### 4.4 Functional Differences Between Serum‐ and Plasma‐Derived EVs

In our dataset, the Plasma_SEC unique set was dominated by translation/proteostasis categories (ribosome, RNA binding, chaperones/proteasome), whereas the Serum_SEC unique set was dominated by immune/adhesion/cytoskeletal/hemostasis pathways. A plausible explanation is that anticoagulated plasma preserves cytosolic ribonucleoprotein complexes and EV subpopulations richer in translational machinery released by circulating blood cells, while serum formation triggers clotting and platelet activation, yielding EVs and co‐isolated proteins with adhesion and coagulation signatures. Recent studies show that extracellular biofluids contain not only membrane‐bound EVs but also small non‐vesicular nanoparticles (e.g., exomeres and supermeres, ~20–50 nm) enriched in proteins and RNAs previously attributed to EVs [63, 64]. Because SEC and density/centrifugation separate particles by biophysical properties rather than membrane status, these non‐vesicular carriers and soluble RNPs may co‐enrich with plasma EV fractions and contribute to ribosome/chaperone/proteasome signals. We therefore interpret the observed plasma “translation/proteostasis” enrichment as reflecting an EV‐enriched nanoparticle continuum, not active translation within EVs. Orthogonal separation and validation approaches will be required to distinguish vesicular from non‐vesicular contributions. In addition, clotting may sequester or degrade free ribonucleoproteins, reducing their carry‐over into serum EV fractions. These functional inferences are derived from unique‐protein sets and should be confirmed with quantitative differential abundance; nevertheless, the pattern is robust across enrichment databases used in our pipeline.

The pathway tilt likely reflects both genuine differences in circulating EV subpopulations and matrix‐dependent co‐enrichment of EV‐adjacent particles. The Plasma_SEC‐unique set (ribosomal proteins, RNA‐binding factors, chaperones, and proteasome components) suggests a “translation/proteostasis” signature consistent with systemic load–related cargo from blood cells and/or co‐isolated RNP‐rich material [65]. This should not be interpreted as active translation within EVs, but rather as preferential capture of proteostasis/RNP‐linked components. In contrast, the Serum_SEC‐unique set was enriched for immune/adhesion/hemostasis categories, which is biologically plausible given clotting‐associated platelet activation and adsorption of complement/coagulation proteins (protein corona), making serum fractions potentially informative for immunothrombotic or vascular phenotypes. Because these enrichments were derived from presence/absence‐defined unique protein sets, they are hypothesis‐generating and should be confirmed using quantitative differential‐abundance analyses and workflows that better control lipoprotein/RNP carry‐over (e.g., ApoA1/ApoB monitoring and orthogonal EV validation).

From a biomarker perspective, matrix choice should therefore follow biology: plasma‐EVs may be better suited to interrogate metabolic/translational or intracellular stress pathways, whereas serum‐EVs may better capture immune activation, coagulation dynamics, and vascular interactions. Cross‐study comparisons must explicitly report and control for the matrix, as the same phenotype can yield divergent biomarker signatures in serum versus plasma. Diagnostic performance is the ultimate benchmark; thus, our study focuses on upstream analytical determinants—matrix selection and isolation workflow—that shape EV yield, marker detectability, and the apparent proteome at the discovery and pre‐validation stage. Uncontrolled co‐isolation and pre‐analytical variability can alter which candidates emerge as “top hits” and undermine reproducibility across cohorts and laboratories. Finally, because bulk blood EVs represent a heterogeneous mixture from multiple tissues, we limit our conclusions to methodological optimization for blood‐based biomarker discovery and system‐level profiling, without implying suitability for tissue‐specific mechanistic analyses.

### 4.5 Pre‐Analytical Variables and Anticoagulant Choice

EDTA‐PTCP (EDTA‐induced pseudothrombocytopenia) illustrates how anticoagulants can reshape hematologic readouts via immune‐mediated platelet clumping [66, 67, 68, 69]. Anticoagulants may also alter EV morphology/function and interfere with downstream assays. Conversely, when blood collection and serum processing are rapid and standardized, platelet activation and release of platelet‐derived vesicles can be minimized. Practically, serum integrates more smoothly with clinical workflows and often shows greater long‐term stability and freeze–thaw tolerance than plasma [65]. Under controlled conditions, serum‐derived EVs can therefore reflect “true” EV signals with fewer matrix‐induced artifacts. Additionally, for MS‐based plasma proteomics, K2‐EDTA plasma is commonly preferred over citrate plasma, consistent with the HUPO Plasma Proteome Project recommendation that EDTA plasma be the preferred sample type for proteomics experiments [70].

### 4.6 Future Perspectives

Beyond veterinary diagnostics, standardized blood‐EV workflows in horses can enable two‐way translation: horses allow repeatable, minimally invasive sampling under naturally occurring high‐intensity exercise and systemic inflammatory challenges, supporting EV‐based monitoring of organism‐level adaptation relevant to sports medicine and immunometabolic research [19]. In human biomedicine, circulating EVs are increasingly explored as liquid‐biopsy substrates in oncology and neurodegeneration, underscoring the need for robust, matrix‐aware pre‐analytics and reproducible isolation pipelines [1, 2, 3]. In this context, our equine benchmarking demonstrates that matrix and workflow materially shape the apparent EV proteome and reproducibility, and that a simpler serum‐SEC workflow can provide a practical, reproducible baseline for discovery and pre‐validation. Importantly, SEC is no longer limited to manual low‐throughput columns: automated/plate‐based SEC and SEC‐FPLC implementations are emerging to support clinical‐scale processing.

Future work should address whether these compositional differences translate into distinct functional properties, for example by testing serum‐ versus plasma‐derived EVs in recipient‐cell assays or in vivo models. Longitudinal studies under controlled physiological and pathological conditions would clarify whether the observed proteomic distinctions are stable features or context‐dependent. Given variability in canonical EV marker detection between matrices, species‐specific reference markers for equine EV studies should be defined based on proteomic evidence rather than extrapolated from human consensus panels. Beyond proteomics, integrating lipidomics and small‐RNA cargo could capture matrix‐specific biology that proteins alone might miss, and orthogonal validation (e.g., immunocapture/flow cytometry, Western blotting of marker panels, immuno‐TEM) would strengthen mechanistic inferences. By contrast, precipitation‐based enrichment is typically non‐selective and may increase co‐recovery of soluble proteins and lipoproteins, which can complicate discovery‐stage interpretation [71]. Notably, SEC‐based plasma EV workflows have been reported to support miRNA quantification and small RNA sequencing when input volume and concentration steps are optimized [42, 72]. Therefore, lack of miRNA data in the present study reflects prioritization of proteomics and per‐animal volume constraints rather than an inherent incompatibility of SEC/UC + SEC with transcriptomics. Integrating lipidomics and small‐RNA profiling with proteomics would help deconvolute EV‐derived signals from lipoprotein carry‐over in equine blood and strengthen mechanistic interpretation.

To strengthen mechanistic inferences and disentangle EV‐associated cargo from co‐isolated nanoparticles in equine blood, future studies should implement orthogonal, targeted validation steps such as TEM (and/or single‐EV immunocapture platforms/flow cytometry) to confirm vesicle identity and the localization of selected equine reference markers, as well as targeted MS using a predefined peptide panel to quantify purity and inter‐workflow reproducibility, and small‐RNA profiling.

### 4.7 Limitations

Limitations include the modest sample size and the absence of functional assays confirming biological roles of identified proteins. However, this cohort size is typical for methodological EV isolation comparisons focused on analytical reproducibility and workflow‐dependent biases rather than clinical performance [29, 71]. Because only healthy horses were included, we did not evaluate diagnostic performance metrics (sensitivity/specificity) or clinical classification; these endpoints will require disease cohorts and targeted validation assays.

Pre‐analytical factors (time to processing, freeze–thaw history) may contribute to variability, and lipoprotein co‐isolation—particularly HDL—cannot be excluded; ApoA1/ApoB proxies were not monitored. Enrichment analyses are additionally constrained by incomplete *Equus caballus* annotations. We also did not quantify total RNA or miRNA cargo, precluding evaluation of SEC versus UC + SEC for transcriptomic endpoints, and the manual mini‐SEC workflow (especially UC) is not optimized for high‐throughput clinical deployment. Future work should therefore incorporate routine QC (ApoA1/ApoB, ISEV/ISMVs negative markers, SEC column reuse metrics) and orthogonal validation (immuno‐TEM, density‐gradient separation, small‐RNA profiling) [73, 74], noting that immuno‐based validation is further limited by the availability and cross‐reactivity of equine‐validated antibodies and supports the need for equine‐adapted marker panels.

We did not compute particle‐to‐protein ratios across all groups because particle concentration (NTA) and total protein yield were not systematically acquired for every matrix × isolation condition. We also did not perform intensity‐based differential proteomics (fold changes) because DDA label‐free on Orbitrap‐based MS intensities are sensitive to non‐random missingness and to isolate yield/loading effects; in this methodological setting, overlap‐based reproducibility metrics (≥ 3/6 and 6/6 core) were considered a more robust basis for comparing workflows. However, as a semi‐quantitative complement, we report per‐run identification depth (median protein identifications and ranges) for each condition, which provides an indicator for analytical depth and reproducibility across workflows.

## 5 Conclusions

We compared EVs from equine serum and plasma isolated by SEC and UC + SEC. SEC outperformed UC + SEC in practicality and reproducibility, and serum_SEC yielded the most consistent proteomes (e.g., higher ≥ 3/6 overlap). In our hands, adding pelleting UC prior to SEC did not improve downstream proteomic readouts and was associated with increased between‐sample variability, arguing against UC as a necessary step for routine equine blood‐EV proteomics under the tested conditions. From a practical standpoint, serum is also the more economical and operationally convenient matrix; therefore, unless anticoagulated plasma is specifically required by the biological question, we recommend serum+SEC as the default for equine blood‐EV proteomics. TEM showed typical small, cup‐shaped vesicles, and NTA indicated a dominant 100–200 nm population, supporting that the isolates represent bona fide EVs.

Proteomically, the matrix‐shaped EV cargo: Plasma_SEC was enriched for translation/proteostasis (ribosome, chaperones, proteasome), while Serum_SEC was enriched for immune/adhesion/cytoskeletal/hemostasis pathways—consistent with greater contributions from circulating blood cells and vascular interactions. Practically, we recommend serum + SEC as a robust default for equine blood‐EV proteomics and biomarker discovery, with strict control of pre‐analytics. Given equine‐specific features (HDL‐rich plasma, protein/lipoprotein corona), studies should justify matrix/method choice, use equine‐appropriate marker panels and negative controls, and report pre‐analytical handling transparently to enable reproducible, translational EV research.

## Author Contributions

Conceptualization, M.Ż., J.O., O.W.‐P., D.M.‐H.; formal analysis, O.W.‐P., D.M.‐H., B.Ś., P.K., J.O. and M.Ż.; investigation, O.W.‐P., D.M.‐H., P.K., J.O. and M.Ż.; writing – original draft preparation, O.W.‐P., D.M.‐H., J.O., P.K. and M.Ż.; writing – review and editing, O.W.‐P., M.Ż., J.Ś., P.K., D.M.‐H., J.O.; visualization, O.W.‐P. and M.Ż.; supervision, O.W.‐P.; project administration, O.W.‐P.; funding acquisition, O.W.‐P. All authors have read and agreed to the published version of the manuscript.

## Funding

This research was funded in part by the National Science Centre, Poland, Sonata 18, No. 2022/47/D/NZ7/01814 (O.W.‐P.) and by the Science Development Fund of the Warsaw University of Life Sciences—SGGW. For open access, the author has applied a CC‐BY public copyright license to any author‐accepted manuscript (AAM) version arising from this submission.

## Ethics Statement

The horses were admitted to the clinic for routine procedures, during which blood sampling was performed as part of standard health examinations. Surplus blood, not required for clinical purposes, was used for the analyses. In accordance with the European Directive 2010/63/EU and Polish regulations on animal experimentation, these procedures qualified as non‐experimental clinical veterinary practices and therefore did not require Ethics Committee approval. Written informed consent for the use of surplus blood in scientific analyses was obtained from owners.

## Consent

The authors have nothing to report.

## Conflicts of Interest

The authors declare no conflicts of interest.

## Supporting information


**Table S1:** Complete proteomics results for EV‐enriched fractions from equine plasma and serum processed with SEC/Ultra‐SEC (24 samples; 4 groups × 6). The XLSX workbook includes: Samples legend (sample mapping), Summary (per‐sample IDs and EV markers: CD9, CD63, CD81, CD82, TSPAN7, TSPAN9, TSG101, PDCD6IP), IDs all (1784 proteins with FDR/q‐value/PEP, peptide/PSM counts, and presence across samples), and group sheets: Plasma_SEC (*n* = 1423), Plasma_Ultra‐SEC (*n* = 591), Serum_SEC (*n* = 877), Serum_Ultra‐SEC (*n* = 874).

**Figure S1:** Top enriched terms (unique sets).

## Data Availability

The mass spectrometry proteomics data have been deposited to the ProteomeXchange Consortium via the PRIDE partner repository under accession PXD068077 (DOI: 10.6019/PXD068077). Processed summary tables are provided as Supporting Information Figures  [Link], [Link] (*OWP_EVs_test_all_noRNA_noWIR_standard_groups.xlsx*).
