# Supplementary figures and images for: Comparative Analysis of Extracellular Vesicle Isolation From Equine Serum and Plasma Using Two Isolation Methods With Structural and Proteomic Validation
Source: FASEB J. 2026 Jan 18;40(2):e71472. doi: 10.1096/fj.202504053R (PMC12813514; doi:10.1096/fj.202504053R)

**A**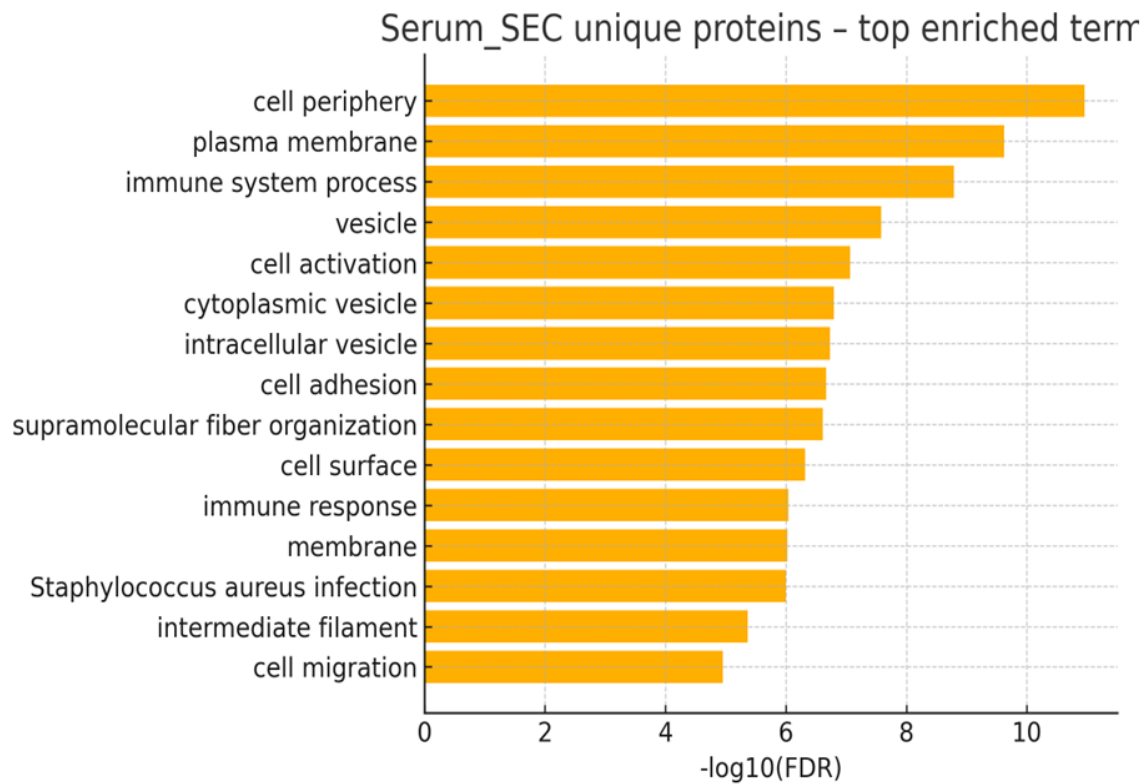**B**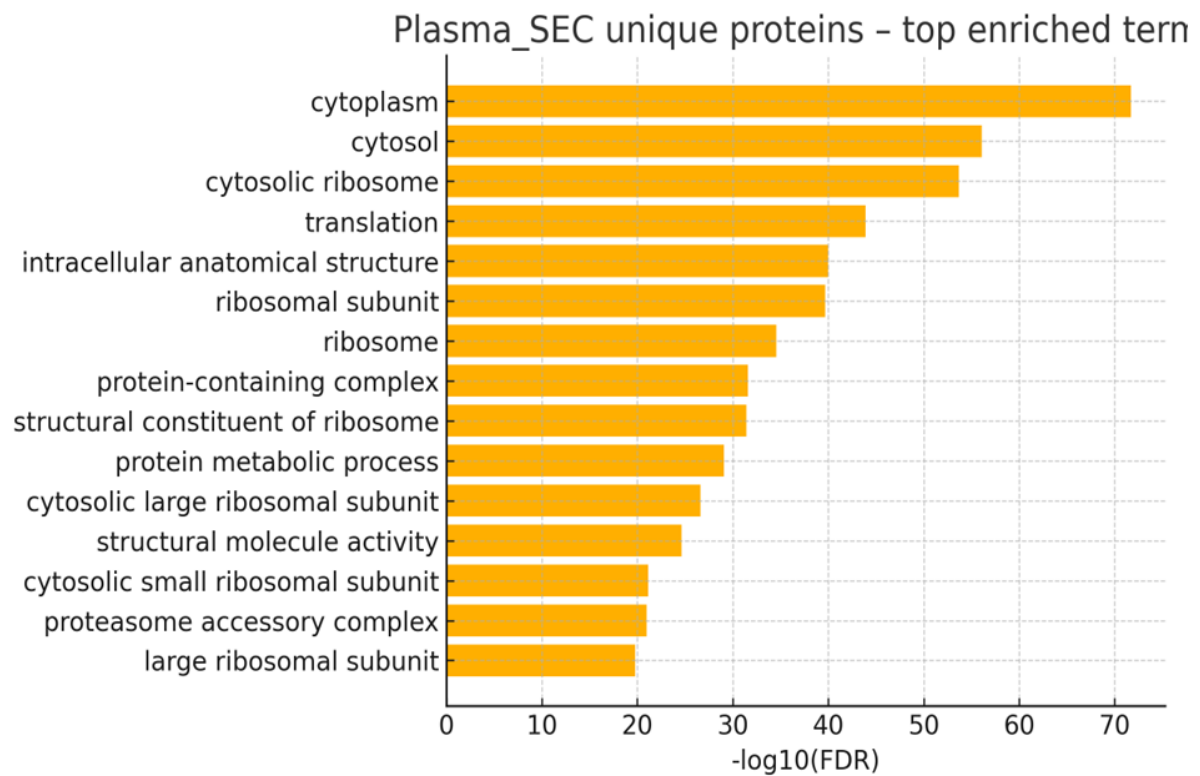

Supplement: Supplementary file 2 — Figure S1: Top enriched terms (unique sets). [file FSB2-40-e71472-s002.pdf]
